# Discharge interventions from inpatient child and adolescent mental health care: a scoping review

**DOI:** 10.1007/s00787-020-01634-0

**Published:** 2020-09-04

**Authors:** A. Chen, C. Dinyarian, F. Inglis, C. Chiasson, Kristin Cleverley

**Affiliations:** 1 Faculty of Medicine, University of Toronto, Toronto, Canada; 2 Lawrence S. Bloomberg Faculty of Nursing, University of Toronto, Toronto, Canada; 3 Centre for Addiction and Mental Health, Toronto, Canada; 4 Department of Psychiatry, Faculty of Medicine, University of Toronto, Toronto, Canada

**Keywords:** Mental health, Discharge, Scoping review, Child, Adolescent

## Abstract

The post-discharge period is an extremely vulnerable period for patients, particularly for those discharged from inpatient children and adolescent mental health services (CAMHS). Poor discharge practices and discontinuity of care can put children and youth at heightened risk for readmission, among other adverse outcomes. However, there is limited understanding of the structure and effectiveness of interventions to facilitate discharges from CAMHS. As such, a scoping review was conducted to identify the literature on discharge interventions. This scoping review aimed to describe key components, designs, and outcomes of existing discharge interventions from CAMHS. Nineteen documents were included in the final review. Discharge interventions were extracted and summarized for pre-discharge, post-discharge, and bridging elements. Results of this scoping review found that intervention elements included aspects of risk assessment, individualized care, discharge preparation, community linkage, psychoeducation, and follow-up support. Reported outcomes of discharge interventions were also extracted and included positive patient and caregiver satisfaction, improved patient health outcomes, and increased cost effectiveness. Literature on discharge interventions from inpatient CAMHS, while variable in structure, consistently underscore the role of such interventions in minimizing patient and family vulnerability post-discharge. However, findings are limited by inadequate reporting and heterogeneity across studies. There is a need for further research into the design, implementation, and evaluation of interventions to support successful discharges from inpatient child and adolescent mental health care.

## Introduction

Discharge from psychiatric inpatient care can be a time of vulnerability for patients due to the complexity of instructions, transitions between care providers, and shifts in responsibility of those involved [1–3]. These risks may be even more pressing for the pediatric age group, a demographic more likely to experience the onset of mental illness than any other age group [4]. In the United States, pediatric mental health admissions have increased approximately 50% in the past fifteen years [5] and a doubling of emergency department visits related to suicidal attempts and suicidal ideation among youth during 2007–2015 [6]. Existing research has shown preventable adverse events, risk of suicidality, and readmissions are heightened during the post-discharge period [2, 7–11]. Inadequate discharge practices can contribute to disjointed care coordination, greater risk of relapse, and poorer patient health outcomes [12].

Inpatient psychiatric admission can pose considerable stress for the patient and their family [13–15], and readmissions can introduce further personal and health care costs [16, 17]. Readmission may reflect the quality of inpatient care, discharge planning, and aftercare provided in the mental health care system [18]. Readmission rates for children and youth have been shown in the literature to range from 12–65% in the year following discharge [19–21]. The 30-day unplanned readmission rate for children with a mental health presentation has been calculated to be higher (8%) for those with a non-mental health presentation (6.2%) [22]. Collectively, these findings suggest an urgent need to examine interventions to reduce readmissions and support youth in the post-discharge period.

Discharge interventions are defined as single or multifaceted interventions involving personal contact between the patient and their care team (i.e. hospital staff, community workers, service providers) that aim to prevent or solve anticipated problems in subsequent outpatient or post-discharge care, facilitate continuity of care, and reduce adverse events post-discharge [23–26]. Discharge interventions have been evaluated to some extent in both child and adult inpatient and outpatient settings [9, 26, 27]. However, the effectiveness of these interventions has not been extensively described or evaluated in the child and adolescent mental health care services (CAMHS) [28]. Systematic reviews examining transitional interventions or discharge planning from inpatient to outpatient settings have limited their inclusion criteria to the adult population (> 18 years old) [29–32] or have focused primarily on interventions taking place in vocational or educational settings [28]. A recent scoping review identified discharge planning as a core component of transitions from CAMHS [33], yet there is limited literature reviewing the components of interventions facilitating successful discharges from inpatient CAMHS and whether current interventions are effective.

To address this gap, this scoping review aims to explore literature on interventions that facilitate discharges from inpatient CAMHS. Specifically, this review will (1) describe the key components and (2) identify outcomes of existing discharge intervention from inpatient CAMHS. Through this review, we hope to identify knowledge gaps in inpatient CAMHS discharge interventions described to-date.

## Methodology

This review follows the scoping review framework outlined by Arksey and O’Malley [34], and further refined by Levac et al. [35]. The PRISMA extension for scoping reviews (PRISMA-Scr) checklist [36] was used in the reporting of the results. The six steps of the framework are further described below.

### Stage 1: identifying the research question

This scoping review aims to explore what is known from the literature about interventions facilitating discharges from inpatient CAMHS. Discharge interventions were defined as interventions at any point of the care pathway that aimed to support patients with anticipated issues once discharged from inpatient services [31, 32, 37]. The research question guiding this scoping review was: what are the discharge components, designs, and outcomes of discharge interventions for children and adolescents (< 18 years old) receiving inpatient mental health care?

### Stage 2: identifying relevant studies

Search strategies were drafted by a research librarian (FI) using subject heading and text word terms based on the key concepts of the research question, and then further refined through discussions with other members of the research team (KC, AC). Terms for the concept of discharge included intervention, aftercare, and transition. Terms for the concept of mental health care included mental health and psychiatric combined with services, recovery and hospital. Terms focusing on youth were also included. Once the initial search was finalized in Medline (Ovid), it was translated for the following additional databases, Embase (Ovid), PscyINFO (Ovid), CINAHL (EBSCO), and Applied Social Sciences Index and Abstracts (ProQuest). The results included all articles published up until the date of the search (May 30, 2019). The full search strategy for Medline is available in Online Resource 1.

A grey literature search was conducted using Google Advanced and the Canadian Agency for Drugs and Technologies in Health search tool, “Grey Matters” [38] using the search terms listed above. Additional studies were identified through hand-searching of reference lists of eligible articles not included or not captured by electronic databases.

The research team came to consensus on inclusion and exclusion criteria for study articles. To be included in the review, studies needed to (1) describe a discharge intervention; (2) be inclusive of a child and adolescent population (< 18 years old) with a primary mental health and/or addiction concern or diagnosis; and (3) be conducted in an inpatient hospital setting. Interventions that were exclusively based in the emergency department or forensic settings were excluded from this review as these settings may offer greater variability in the admission process. For instance, the majority of pediatric mental health emergency department visits end in discharge rather than admissions [39]. Due to resource constraints, documents that were not available in English were also excluded.

### Stage 3: study selection

#### Title and abstract review

Article screening was conducted in a two-stage process. Two researchers (AC and CD) first pilot tested the eligibility criteria on a random sample of 30 articles. Discrepancies were identified and minor changes were made to the eligibility criteria to ensure clarity and consistency in the review process. Using Covidence software [35], two reviewers (AC, CD) then independently evaluated the title and abstracts to the inclusion criteria, and a third reviewer (KC) was consulted for conflict resolution. All study methodologies (experimental, quasi-experimental, observational studies) and non-research studies (review articles, dissertations documents, conference papers) that described discharge interventions for inpatient CAMHS were included. Title and abstracts of gray literature were not reviewed at this stage due to lack of abstracts and the length of documents. Studies focusing on populations ≥ 18 years with a primary diagnosis of autism spectrum disorders, developmental disorders, or intellectual disorders and without a primary diagnosis of a mental illness were excluded. While often concurrent with mental disorders, the unique needs of this population and how they may present for admission may require interventions that are distinct from those with a primary mental illness diagnosis [40, 41].

#### Full-text review

Full-text review of the selected peer-review articles and gray literature documents were subsequently screened by reviewers AC and CD for eligibility using the inclusion criteria. KC was consulted in the event of a conflict for resolution. Conference abstracts and posters were excluded given insufficient detail provided for extraction.

### Stage 4: charting the data

Two reviewers (AC, CD) developed a data-charting form to determine the data to extract with additional review by KC. This data-charting form was updated in an iterative manner. Data extraction included document characteristics (i.e., author, title, document type, country of origin, setting), research question, aims and objectives, sample size and description, study design, methods, intervention service providers, description of intervention and its components, outcomes, evaluative measures, results, and future directions. For non-studies, it was specified where some categories were not applicable. The reviewers extracted data from two randomly selected articles to ensure reliability of the content entered in the data-charting form. The remaining articles were divided, and the reviewers performed independent data extraction.

Using the scoping review’s research question as a guide, a directed content analysis approach was used to organize and analyze the data [42]. During this iterative process, duplicate components or characteristics were removed, and categories with the same content were merged. This process facilitated the process of identification and classification of discharge intervention components.

### Stage 5: collating, summarizing, and reporting results

To provide an overview of the target population, intervention provided, and outcomes, a narrative synthesis was conducted, a process which entailed reviewing the documents and components of discharge interventions described. Arskey and O’Malley’s [34] descriptive-analytic approach was used to help identify patterns and themes among discharge intervention components. The research team engaged in an iterative process of reviewing the interventions, removing duplicate components or characteristics, and collapsing content with similar components. The included documents’ methodological quality or risk of bias were not critically assessed given the aims of this scoping review.

To address our first research question regarding intervention structure, this paper adapted a taxonomy for discharge interventions originally described by Hansen et al. [43] and since replicated in other peer-reviewed articles examining discharge interventions [24, 44]. Hansen et al. [43] described single interventions which fell into three temporal categories: pre-discharge (interventions taking place prior to hospital discharge), post-discharge (interventions supporting patients’ post-hospital discharge), and bridging (interventions that begin prior to discharge and support patients through different care settings). This taxonomy was applied to organize the intervention components of this scoping review along a temporal continuum.

To address the intervention components themselves, the NICE guideline was utilized. NICE highlights a pathway for discharge from inpatient mental health services to community or care home support [45]. The pathway highlighted seven components: caregiver and patient involvement, discharge planning, psychological interventions, peer support consideration, planning of care to support discharge, follow-up support, and reduction of readmissions. These NICE principles were reviewed by the research team and used as an overarching framework to identify and organize components of the discharge interventions in this scoping review.

Outcomes reported by the papers identified were analyzed and grouped according to the Triple Aim Framework. This framework was developed by the Institute for Healthcare Improvement as an approach to optimizing health system performance [46]. The framework is based on the following three aims: (1) improving the individual experience of care; (2) improving the health of populations; and (3) reducing per capita costs of care for populations [46].

## Results

**Fig. 1:**
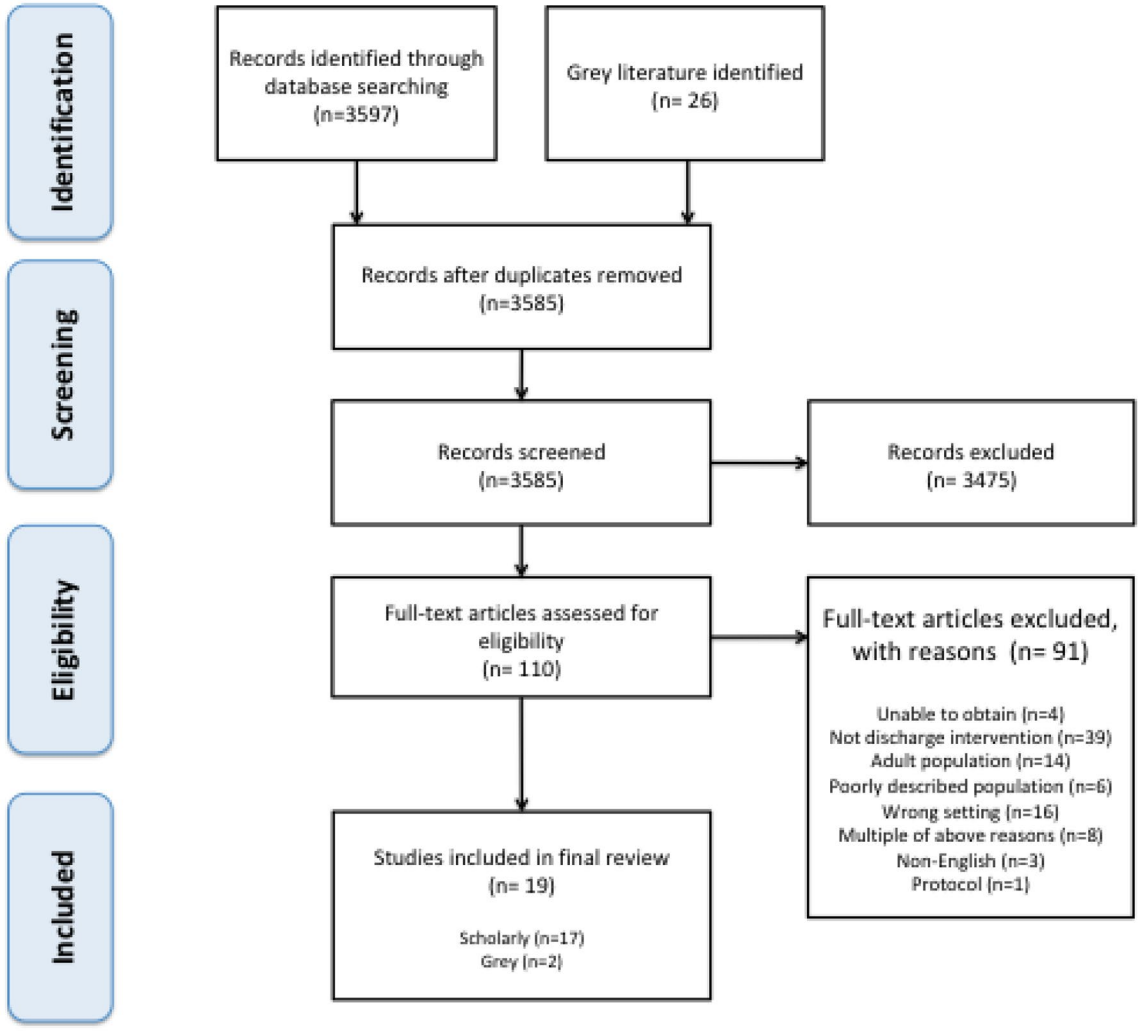
PRISMA flow chart of search results

Database and grey literature searches resulted in a total of 3597 scholarly titles and 26 documents. After removing duplicates, 3585 titles and abstracts were screened, for which 110 documents met criteria for full-text review. Figure 1 displays the screening and exclusion process in greater detail. In total, 17 scholarly articles and two grey literature documents, consisting of a published presentation and dissertation, were included and eligible for extraction.

Table 1 features the extracted characteristics of documents included for final review. Articles were published between 1972 and 2018, with 37% (*n* = 7) of papers published prior to 2000. The majority of articles (84%) were published in North America (nine from USA, seven from Canada), and the remaining three articles were from New Zealand, Germany, and the UK. Of the 17 academic articles included, most (63%) were descriptive reports. There was significant clinical heterogeneity reflected among studies: there were three mixed-method studies, two randomized controlled trials, one feasibility study, and one validation study. The majority of articles included a broad spectrum of mental health conditions, including psychotic, bipolar, depressive, anxiety, substance-related and addictive, personality, and conduct disorders. These discharge interventions predominately took place in child and adolescent psychiatric units, with a few studies involving the emergency department, mobile clinics, schools and/or community agencies.

**Table 1:** Characteristics of included documents

Author, year, Reference	Publication type	Country	Sample size (*n*)^a^	Population	Study design	Setting	Service provider
Baker et al., 2017, [47]	Presentation	Canada	127	13–17 years old Symptoms of complex mental illness (anxiety, depression, psychosis and/or emotional dysregulation)	Program description and evaluation	Referrals across 3 sites, settings include crisis services, mobile teams, hospital, inpatient, urgent care, ED	Clinical team involving counsellors, mental health nurse, public health nurse, occupational therapist, psychology/psychiatry, coordinator, volunteers
Bobier et al., 2009, [57]	Journal article	New Zealand	16	16–18 years old Severe psychiatric disorder; excluded conduct disorder or substance use disorder unless acute axis I comorbid psychiatric disorder	Cross-sectional; mixed-methods	Youth inpatient unit at tertiary mental health facility	Case manager or primary nurse, input received from clinical team (nursing staff, allied health professionals, consultant psychiatrist, medical officer)
Boege et al., 2015, [60]	Journal article	Germany	100	5–17 years old Psychiatric diagnosis at admission as defined by International Classification of Diseases-10th Revision (ICD-10)	RCT	Child and adolescent psychiatry inpatient setting	Inpatient hospital team, child/adolescent psychiatrist, cooperation with social services, schools, physicians
Cameron et al., 2007, [50]	Journal article	Canada	17	13–18 years old (mean age 15.8) Mood disorders, psychosis, pervasive developmental disorders, behavioral issues, eating disorders, suicidal ideation, substance misuse, attachment disorders, personality disorders	Descriptive, mixed data Program evaluation	Adolescent inpatient psychiatry unit and adolescent residential treatment center	Clinical liaison nurse
Chiappetta et al., 2018, [56]	Journal article	USA	111	Mean age 15.1 years old Primary diagnoses of mood disorders, bipolar disorder, attention-deficit/hyperactivity disorder, psychotic disorders, oppositional defiant disorder; multiple concurrent primary diagnoses	Descriptive Program evaluation	Child and adolescent units at urban inpatient psychiatric hospital	Nurse
Cleverley et al., 2018, [58]	Journal article	Canada	N/A	12–18 years old Multiple mental health and developmental disabilities	Report Program evaluation	Inpatient mental health, outpatient unit, day hospital units, community mental health centers	Transitional support services therapist/transition support workers
Doherty et al., 1987, [63]	Journal article	USA	212	Preschool-16 years old Psychosis, suicidal behaviors, aggression towards self, conduct problems	Descriptive	Child psychiatric unit in medical center	Child psychiatrist-director, nursing coordinator, 16 full-time nursing and child milieu staff, social worker-family therapist, expressive therapist, child development specialist-educator. Part-time services provided by pediatrician, psychologist, occupational therapist, administrator
Drell, 2006, [48]	Journal article (innovation column)	USA	80	Not specified	Descriptive	Child and adolescent psychiatry unit	Social work supervisor, social workers, psychiatric aide
Furedy et al., 1977, [51]	Journal article	USA	672	Teenage-70 years old Schizophrenia, psychotic/affective disorders, personality disorder, neurotic, classified ‘other’ disorders	Descriptive	University of Wisconsin Medical Centre	Nursing staff
Gregory et al., 2017, [55]	Journal article	Canada	76	< 18 years	Feasibility study	Child and adolescent inpatient unit	Unit staff (nurses or child and youth counsellors)
Hennessy, 2018, [64]	Dissertation	USA	34	10–24 years old	Mixed methods pilot study	Inpatient psychiatric facility)	Aftercare coordinator
Leung, 1984, [59]	Brief report	Canada	96	2.5–14 years old	Follow-up study	Child psychiatric unit	Social worker and head nurse
Lurie and Ron, 1972, [52]	Journal article	USA	Unspecified	16–25 years old	Descriptive/program evaluation	Community center	Unspecified
Ougrin et al., 2018, [65]	Journal article	UK	108	12–18 years old	RCT	Psychiatric inpatient in the South London and Maudsley NHS Foundation Trust	Each team included one consultant child and adolescent psychiatrist, one administrator, two to four practitioners with nursing backgrounds (full-time equivalents), and two to four clinical support workers (full-time equivalents)
Roy and Helt, 1989, [61]	Clinical forum	USA	Unspecified	Pediatric population, age unspecified	Descriptive	Inpatient psychiatric unit	Clinical specialist leads the parent group. A staff clinician leads the children’s group
Stelzer and Elliott, 1990, [62]	Journal article	Canada	200 patients admitted a year	Ages 5–17 years old	Descriptive	Emergency department and other wards of the Children's Hospital, several different community agencies, the school system, family physicians, and self-referrals	Senior social worker and psychiatrist conduct weekly discharge meetings, trainees are occasionally present. Occupational therapist conducts weekly social skills group for children
Wasylenki et al., 1981, [49]	Journal article	Canada	45	Patients (1977–1979), ages 16–70 years old; mean age = 31 years		University psychiatric hospital in large metropolitan area	Core personnel: psychiatrist director, psychiatric nurse coordinator, psychiatric resident Secondary personnel: liaison representatives from community agencies, public health nurse, hospital's home care coordinator, community resources consultant, a member of the hospital's rehabilitation services department Tertiary personnel: community workers with no formal affiliation with the hospital
Weiss et al., 2015, [53]	Journal article	USA	Unspecified	School-aged youth	Descriptive	Classroom	Transition Team made of Family Connector and School Transition Specialist
White et al., 2006, [54]	Journal article	USA	99	Adolescents; sample from October 2003–Nov 2005 period	Descriptive; longitudinal study	Classroom	Clinician coordinators (i.e., two master's-level social workers)

^a^Sample size refers to the population of interest for our research question

### Discharge intervention structures

Discharge interventions reported in the reviewed literature were varied in structure, owing to a lack of standardization of the terms “discharge intervention” and the diversity in settings and populations across projects. Most discharge interventions (*n* = 9) were structured as a standalone program [47–54]. These programs were longitudinal, multicomponent, and often featured a multidisciplinary team that was responsible for supporting patient discharge. Four programs described bridging aspects with case management, clinical services, discharge planning and referrals to outpatient community resources to maintain continuity of care for the patient [47–50]. Two programs supporting patients in maintaining clinical stability, improving family functioning, and building vocational and social skills through therapy and counselling [51, 52]. Another two papers described programs which provided care coordination between the hospital, family, and school to support the child’s transition back to school after discharge [53, 54].

Other discharge interventions (*n* = 3) described discharge tools or strategies for professionals to facilitate the discharge process. These interventions included feasibility of a safety planning smartphone application [55], motivational interviewing at the time of discharge to identify barriers to attending outpatient appointments [56], and narrative discharge letters, collaboratively prepared by the health care team and patient to facilitate reflection and communication [57].

The roles of different health professionals in the discharge process were also highlighted in the reviewed literature. For example, some articles (*n* = 2) described a designated role or position that provided the discharge intervention. Cleverley et al. reviewed the role of Transition Support Workers (TSW) in the transition from hospital to community [58]. TSWs function as case managers, assist with discharge planning, system navigation, individual and family therapy, and client advocacy [58]. Leung described a similar role, the Senior Therapist, who held responsibilities in case management and facilitating family interviews, therapeutic sessions with the patient and crisis intervention, and arranging follow-up services [59].

### Question 1: key components of discharge interventions

Publications were reviewed for commonalities in components. Based on the interventions classified in each temporal category of Hansen and al.’s taxonomy, five papers were classified as pre-discharge interventions [48, 55–57, 59], four were post-discharge interventions [52, 60–62], and ten were bridging interventions [33, 47, 49–51, 53, 54, 63–65] (see Table 2)*.* Core components of the interventions (see Table 3), described further below, were identified as: (1) risk screening and assessment, (2) individualized care, (3) client discharge preparation, (4) community linkage, (5) psychoeducation, and (6) follow-up support.

**Table 2:** Discharge interventions described in included studies, organized by Hansen et al. taxonomy

Author, Year, Reference	Intervention components	Pre-discharge interventions	Post-discharge interventions	Bridging interventions
Programs (*n* = 9)				
Baker et al., 2017, [47]	Case management Clinical services (therapy, counselling, medication management, health promotion/prevention, occupational therapy, group therapy for skill building) Patient discharge goal-setting goals Discharge planning and referrals for ongoing services			Multicomponent program: case management, clinical services, discharge planning with referrals for outpatient services for continuity
Cameron et al., 2007, [50]	Clinical Liaison Nurse who helps with cross program communication, provides mental health services Case management Connecting with community services			Program: health care professional (clinical liaison nurse) connects adolescents with services in the community
Drell, 2006, [48]	Discharge planning—includes "map for services" Clinical (hospital care) services available if needed			Transition program: individualized discharge plan, facilitating community supports, with continuity of provider (social work supervisor)
Furedy et al., 1977, [51]	Discharge planning Formal therapy, group discussions Skill building support Encouragement of community resources Recreational and social activities			Transitional program occurring post-discharge to build skills and assist patients with post-discharge problems
Lurie and Ron, 1972, [52]	Group Counselling for post-hospital adjustment Activity-oriented self-help groups Vocational counselling Crisis intervention		Program: counselling and self-help groups for patients post-discharge	
Roy and Helt, 1989, [61]	Parent Group: skill-building, problem solving, education Children's group: problem solving, social and behavioral skill building		Skill building groups for parents and children	
Wasylenki et al., 1981, [49]	Community worker—maintains contact with patient during holding period and connects patients with aftercare services			Transitional program: community worker maintains ongoing contact with patient throughout discharge process and connects with aftercare services
Weiss et al., 2015, [53]	Consultation with school and hospital staff Transition support plan Family/caregiver education and feedback to family Peer support Connection to community services			Family Connection and School Transition Specialist: connects with patients, school, and hospital staff, assisting patients in transition from hospital to school and connecting to community services
White et al., 2006, [54]	Case management Student and family counselling Facilitating communication with school, health providers, other agencies Parent support and psychoeducation group			Transition program: case management, counselling, community liaison to prepare and support the patient for return to school
Single intervention discharge tools (*n* = 4)				
Bobier et al., 2009, [57]	Narrative discharge letter: written in collaboration with patient	Single discharge planning intervention: narrative discharge letter writing		
Chiappetta et al., 2018, [56]	Nurse-administered MI discharge process; educational packets for families	Motivational interviewing at time of discharge and educational packets for families		
Gregory et al., 2017, [55]	Smartphone application for safety planning and direction to resources	Discharge tool implemented at the time of discharge: smartphone application for safety planning and direction to resources		
Hennessy, 2018, [64]	Discharge planning Patient education about illness/resources Coordination of follow-up post-discharge for patients identified as high-risk	Discharge planning tool and patient education rand resources		Designated healthcare professional (aftercare coordinator): assessed discharge preparedness prior to discharge with follow-up of high-risk patients
Models (*n* = 4)				
Boege et al., 2015, [60]	Early discharge Home treatment with case management, individual therapy, family therapy Clinical elements (day hospital, hospital schooling) Crisis management Cooperation with social services, schools, physicians		Hospitalization limited treatment: clinical elements continued post-discharge with home treatment and hospital, crisis management	
Doherty et al., 1987, [63]	Case management Linking with community resources Advocacy strategies by supporting parents to attend planning meetings Family milieu therapy and other family-oriented technique Use of therapeutic leaves of absences			Treatment model featuring case management, community outreach, centered discharge instruction, outpatient support, aiming to limit hospitalization length of stay
Stelzer and Elliott, 1990, [62]	Follow-up meetings with family to discuss problems arisen post-discharge Social skills group		Follow-up meetings with families discussing problems post-discharge and social skills groups for patients	
Ougrin et al., 2018, [65]	Early Supported Discharge Service (alternative to extended inpatient care) Case management Community treatment and day care in hospital with medical/psychological services School reintegration support			Supported discharge service: continuity of care through earlier discharge and continued day treatment with clinical services
Discharge professional role (*n* = 2)				
Cleverley et al., 2018, [58]	Role: transitional worker/therapist Discharge planning Case management/system navigation Clinical: individual and family therapy Client advocacy Trauma/psychological consult			Designated transitional worker leading case management, system navigation, providing continuity through transition from hospital to community
Leung, 1984, [59]	Role: senior therapist Case management Family interviews Therapeutic sessions with patient Crisis intervention and follow-up services	Designated health professional (senior therapist): case management, therapeutic client and family sessions, discharge planning

**Table 3:** Discharge interventions described in included studies, organized by NICE discharge pathway

Intervention core components	Number of studies included (*N*)	References
Risk screening and assessment	*N* = 5	Drell [48] Wasylenki et al. [49] Boege et al. [60] Stelzer and Elliott [62] Hennessy [64]
Individualized care	*N* = 10	Baker et al. [47] Drell [48] Cameron et al. [50] White et al. [54] Cleverley et al. [58] Leung [59] Boege et al. [60] Doherty et al. [63] Hennessy [64] Ougrin et al. [65]
Client discharge preparation	*N* = 7	Gregory et al. [55] Chiappetta et al. [57] Bobier et al. [57] Cleverley et al. [58] Roy and Helt [61] Doherty et al. [63] Hennessy [64]
Community linkage	*N* = 8	Baker et al. [47] Wasylenki et al. [49] Cameron et al. [50] Furedy et al. [51] Lurie and Ron [52] Weiss et al. [53] Cleverley et al. [58] Doherty et al. [63]
Psychoeducation	*N* = 8	Baker et al. [47] Weiss et al. [53] White et al. [54] Chiappetta et al. [56] Cleverley et al. [58] Roy and Helt [61] Doherty et al. [63] Hennessy [64]
Follow-up support	*N* = 7	Wasylenki et al. [49] Furedy et al. [51] Lurie and Ron [52] White et al. [54] Leung [59] Stelzer and Elliott [62] Hennessy [64]

### Risk screening and assessment

Several interventions (*n* = 5) described an intake process with discussions at time of admission that allowed the health care team to gather information on the accommodations necessary for the patients during hospitalization and post-discharge. This meeting was described as a part of the intake of clients or patients in the intervention, with the goal of reducing readmission [60]. Meetings covered topics such as client issues and conflicts with family or other community agencies [62] and their “medical, therapeutic, vocational, social, recreational, and housing needs” [49]. Stelzer et al. described an interdisciplinary intake meeting exploring client issues and conflicts [62]. The assessment process in the Transition Program described by Drell et al. was key to other interventions to assess suitability for early discharge, providing adequate community support [48]. Other interventions were designed such that participants previously identified as a high risk would be referred as a candidate for the program [64]. Patients’ needs were then reassessed in an iterative manner throughout the course of intervention [48, 60, 62, 64].

### Individualized care

Structuring the intervention to the needs of the patient was discussed as a means of encouraging rapport and adherence. Many authors (*n* = 5) discussed the task of case management in tailoring treatment and discharge plan to the patients’ needs [50, 58–60, 65]. Interventions included personalized goal-setting tools and identifying barriers to services that would allow the healthcare team to then determine safety plans [64], work with them toward identified goals [54], or possible solutions in the discharge plan [47, 48, 58]. For the models that focused on early discharge, individualized treatment plans were developed for the patients in collaboration with other health professionals and services [60, 63]. Two studies included elements of advocacy to ensure patients had appropriate and preferred care for that patient and family [58, 63]. Ensuring smaller patient caseloads per staff allowed for greater flexibility in tailored services.

### Client discharge preparation

Numerous papers (*n* = 7) described an element of discharge planning, which is defined as the coordinated process of supporting the client from hospital and into the community [27]. The approach to discharging the client in these interventions was presented in a variety of different forms. Bobier et al. described a narrative discharge letter that the team wrote in collaboration with the client for its therapeutic value [57]. Roles such as the Transition Support Worker or community-based case manager worked within the care team to spearhead the discharge planning process [58, 63]. Chiappetta et al. described an intervention that used motivational interviewing to explore post-discharge obstacles and solutions and allowed for better individualization of resources [56]. Similarly, other tools, such as the Preparedness Assessment Tool, has been used in monitoring preparedness and informing post-discharge coordination [64]. This discharge process could also involve multidisciplinary input and welcomed the involvement of the family and client. Roy and Helt described organized opportunities for patients and family members to raise issues and concerns that they faced during the post-discharge period and could cover several broad topics, such as health, recreational activities, basic needs, finances, and employment [61]. Gregory et al. described a smartphone application which reviews safety planning around time of discharge [55].

### Community linkage

Many articles (*n* = 8) emphasized the involvement of community agencies or services within their intervention. Several authors referenced that health professionals would assist the clients or patients in identifying resources and making referrals to community agencies [47, 50, 58, 63]. Furedy et al. and Lurie et al. integrated exposure to community services, vocational counselling, and supporting patients in building skills that would support them in post-discharge community integration [51, 52]. Wasylenki et al. also described the maintenance of those relationships with the community, even keeping a list of doctors and psychiatrists in caring for them [49]. Weiss et al. described a “Connect and Reflect” program where adults may consistently check in with a student recently discharged to promote greater connectedness in school [53].

### Psychoeducation

A number of interventions (*n* = 8) also included psychoeducation for both the patient and the parent to ensure that they were adequately supporting post-discharge. Services for clients were focused on building coping strategies, managing emotions [47], and self-management skills [61]. Several interventions included parental involvement, and interventions delivered psychoeducation through parent psychotherapy groups [53, 54, 56, 64], peer-to-peer support [47, 61], supportive therapy [58, 63], problem-solving and awareness [61].

### Follow-up support

Lastly, seven post-discharge or bridging interventions described an element of follow-up support to ensure continuity of benefits the intervention provided and reduce readmission. Furedy et al. described weekly team meetings discussing the progress on the problems they had identified during admission and crisis [51]. Hennessy also arranged post-discharge care through two in-person meetings and phone calls, each time monitoring the patient’s hope and resources [64]. Leung described crisis intervention as a follow-up service for the patient and family [59]. Therapeutic groups were held by Lurie et al. and Stelzer et al. weekly to discuss post-hospital adjustment [52, 62]. Phone calls were also another means of following-up with patients [49, 62]. White used face-to-face therapy, group support, phone calls, and continuity of provider to ensure patient follow-up [54].

### Question 2: discharge intervention outcomes

Outcomes were classified as described in the methods section using the Triple Aim Framework [46] (see Table 4).

**Table 4:** Summary of discharge intervention outcomes and results by Triple Aim Framework: patient experience, population heath, system costs

Author, year, Reference	Patient experience (*n* = 13)^a^	Population health (*n* = 13)^a^	System costs (*n* = 7)^a^
Baker et al., 2017, [47]		(1) Multidimensional Anxiety Scale for Children 2nd Edition-Self Report (MASC-2-SR): reductions in overall anxiety, worry, performance fears, OCD-related symptoms, and physical sensations/panic (2) Children's Depression Inventory 2nd Edition: Self-Report (CDI-2:SR): reductions in overall depressive symptoms and emotional problems (3) Adolescent Alcohol and Drug Involvement (AADIS) and Health of the Nation Outcome Scales for Children and Adolescents (HoNOSCA): reductions in numbers of clients meeting Youth Services Bureau of Ottawa criteria for “high-risk” designation (4) Increased number of clients involved in services post-intervention (5) Increased number of referrals to program or to community services (6) Decreased number of readmissions in ER	
Bobier et al., 2009, [57]	(1) Qualitative: positive feedback regarding utility and comprehensibility of information, format, language of letter, facilitated patient empowerment, enjoyment in facilitating working together with youth and inpatient services		
Boege et al., 2015, [60]		(1) Children's Global Assessment Scale (CGAS): improved clinical functioning in both intervention and control groups and significant within-group comparisons between T1 and T2	(1) Increased cost effectiveness in intervention group (factoring in length of stay, costs of hospitalization, therapy, services) (2) Decreased inpatient length of stay
Cameron et al., 2007, [50]	(1) Qualitative: increased satisfaction in program, positive patient and provider experience, describing improved continuity of care and transition experience, positive feedback on clinical liaison nurse role	(1) Decreased number of readmissions to YAP and other adult inpatient units (2) Decreased number of emergency visits	(1) Decreased length of stay in inpatient unit
Chiappetta et al., 2018, [56]		(1) Increase of 10% in attendance at scheduled follow-up appointments (2) Increase in patient-reported likelihood of attending follow-up appointments (3) Decrease of 4% in cancellations and no-show appointments	(1) Length of stay in hospital shorter in intervention group but not statistically significant
Cleverley et al., 2018, [58]	(1) Qualitative: internal evaluation of patient satisfaction and positive patient feedback	(1) Decreased number of hospitalizations	
Doherty et al., 1987, [63]		(1) Post-discharge placement of patient (re-hospitalized, discharged, foster placement): 22% discharged home as trial basis, 15% required long-term treatment after time-limited hospitalization (hospital or residential); 8% in foster placement due to poor home environment, (2) Percentage of patients who completed treatment: 96%	(1) Length of stay: average of 28 days, ranging from 1–71 days
Drell, 2006, [48]	(1) Increase in patient compliance (2) High family satisfaction *Outcome measures unspecified*	(1) Readmission rates: low (2) Number of community referrals: increased	(1) Length of stay for initial and subsequent admissions decreased
Furedy et al., 1977, [51]	(1) Qualitative: favorable comments from patients about the program and staff observed changes in patient and family behaviors	(1) Readmissions to hospital: no hospitalizations among patients treated in first six months of the transitional-care program	
Gregory et al., 2017, [55]	(1) Uptake of the application (18% downloaded, 76% had interest or intent in downloading the smartphone application)		
Hennessy, 2018, [64]	(1) Preparedness Assessment Tool (PAT): collected average patient preparedness scores over time and scored by averaging patient’s feelings of hope, adequacy of support, self-management (2) Qualitative: positive feedback on PAT tool utility and feasibility; finding it to be user-friendly, efficient at predicting patient preparedness and helpful for personalizing care, guiding interventions, increasing patient collaboration, and monitor progress	(1) Readmission to hospital within 30, 60, 90 days: patients with follow-up visits with the Aftercare Coordinator (AC) were readmitted fewer times within the study period and within 30–90 days post-discharge (2) Number of post-discharge visits with the AC; found to be inversely related to the number of adverse events (3) Patient preparedness not found to be statistically significant in being related to adverse events and readmission	
Leung, 1984, [59]	(1) Qualitative: positive feedback from parents regarding effectiveness of services (2) Qualitative: parents raised concerns regarding follow-up care and inadequate community resources		
Lurie and Ron, 1972, [52]	(1) Case notes and staff ratings of status-role adjustment to work-school, peers, family communication, family adjustment *Outcome results not described*		
Ougrin et al., 2018, [65]	(1) Child and Adolescent Service Experience: similar service satisfaction among both discharge intervention group and usual group	(1) Children’s Global Assessment Scale (CGAS): clinical functioning was similar in the intervention and usual care group at baseline and 6 months follow-up (2) Self-Harm Questionnaire: patients in intervention group were less likely to report multiple (≥ 5) episodes of self-harm compared to usual care group (3) Reintegration to community schools (measured by attendance at community school, number of days not in employment, education, or training): improved reintegration in intervention group	(1) Cost-effectiveness (analyzed through acceptability curves based on CGAS and QALY): intervention group has at least a 50% probability of being cost-effective compared with usual care, irrespective of the measure used and willingness to pay for outcome improvements (2) Time in psychiatric inpatient treatment (measured by occupied bed-days): reduced bed usage at 6 months’ follow-up in intervention group
Roy and Helt, 1989, [61]	(1) Qualitative feedback from parents regarding benefits of post-discharge groups: increased self-awareness, ideas to approach issues, feelings of hope and self-esteem (2) Qualitative feedback from children regarding benefits of post-discharge groups: positive reminders to work on each other, positive peer pressure, improved self-esteem		
Stelzer and Elliott, 1990, [62]	(1) Satisfaction scales: high degree of satisfaction by both parents and children/adolescents	(1) Readmission rates: 8.7% of the yearly study population readmitted	
Wasylenki et al., 1981, [49]		(1) Readmission rate: aftercare program was effective in limiting the number of readmissions during its first two years to 20% (2) Ability to arrange community placement for patients: *n* = 6 (13%) unable to be placed	
Weiss et al., 2015, [53]	(1) Caregiver strain: diminished (2) Caregiver empowerment: increased (3) Caregiver satisfaction with the program: high		
White et al., 2006, [54]		(1) Number of students that remained in community for length of program period: 88 (2) Number of students re-hospitalized: 11 (3) Number of students attending school regularly in follow-up sample (88%) or receiving home tutoring (12%) (4) Child and Adolescent Functional Assessment Scale (CAFAS): decreased score from admission to three-month follow-up—significant improvement in students’ functioning status	(1) Length of involvement in the program: 2–20 weeks (2) Hours of care coordination required: 21 h on average

^a^Refers to the number of separate studies with these outcome categories

### Patient experience

Most studies (*n* = 9) used qualitative measures to capture patient, family, or provider satisfaction of the intervention itself [48, 50, 51, 53, 57–59, 62, 65]. Bobier et al. captured specific feedback regarding the format and utility of their letter intervention [57]. Their study asked youth, “What did you think about the amount of information packed in the letter?” and “Was the language and style of the letter useful for you?” Articles commented on the positive feedback received from participants on the intervention’s format or delivery [57], increased socialization and cohesion among participants [52], and overall positive reception of the interventions from the participant and/or their parent [48, 58, 59, 62, 65].

Other studies used quantitative measures to assess the uptake of an intervention into clinical practice or a patient’s preparation around discharge. Hennessy explored the potential association of a patient’s preparedness at time of discharge with future readmission through a Preparedness Assessment Tool [64]. A feasibility study by Gregory et al. measured the prevalence of smartphone ownership and the youths’ interest in downloading the ‘Be Safe’ smartphone application [55]. Patient compliance with follow-up outpatient appointments following the interventions was another captured outcome, suggesting effectiveness of this intervention in facilitating outpatient care and patient motivation to attend appointments [48, 56]. Furedy et al. used case notes, patients’ ratings, and staff observations of behavioral changes to address suicidal or assaultive behavior to demonstrate positive impact of the behavior modifications in the interventions [51].

### Population health

Population health outcomes specifically related to adherence to care (including disease-specific outcomes), patient-reported health and quality of life, and self-care skills. Such outcomes were captured in thirteen studies. Baker et al. used the Multidimensional Anxiety Scale for Children (MASC-2), Children's Depression Inventory Self-Report (CDI-2 SR), Adolescent Alcohol and Drug Involvement Scale (AADIS), and the Health of the Nation Outcome Scales for Children and Adolescent (HoNOSCA), and preliminary results of these measures showed reductions in overall anxiety, obsessive compulsive disorder-related symptoms, panic, depressive symptoms, and reductions in the number of clients meeting the organization’s high-risk criteria [47]. Ougrin et al. utilized the Strengths and Difficulties Questionnaire (SDQ), the Self-Harm questionnaire, the Children’s Global Assessment Scale (CGAS), and Goal Attainment Score (GAS), with no significant differences found in symptom and functioning outcomes between the discharge intervention and usual care groups [65]. Boege et al. also used the CGAS measure and found increased scores in both intervention and control groups, with significant within-group comparisons CGAS scores, indicating improvement in patients’ clinical level of functioning before and after the discharge intervention [60]. The discharge intervention described by White et al. also resulted in improved clinical functioning in their intervention focused on improving CAMHS inpatient discharges among youth re-entering intensive school services, as measured by the Child and Adolescent Functional Assessment Scale (CAFAS) [54].

Effectiveness of discharge interventions were assessed through a number of outcome indicators, including completion of intervention [63], number of community referrals or placements made [48, 49], involvement in post-discharge services or community [47, 56, 64, 65], emergency department visits or readmission [47–51, 58, 62–64], and number of other adverse effects, such as medication side effects, school or occupational disciplinary action, risky and impulsive behaviors, and encounters with law enforcement [64]. Results of interventions demonstrated increased number of patients involved in post-discharge services [47, 63, 64], decreased emergency department readmissions [47], increased patients having a planned post-discharge follow-up [56] and increased number of patients with a community referral [47–49]. In the time-limited hospitalization model described by Roy and al., the majority (96%) of patients successfully completed the intervention, with only 2% of participants readmitted to the unit [63]. Ougrin et al. evaluated reintegration into community through post-discharge outcomes of study participants, such as increased attendance in the community, decreased unemployed days, and increased days spent in education, or in training [65].

### System cost

The third aim outlines costs to the healthcare system. Cost outcomes of discharge interventions were captured as calculations of cost effectiveness [65], costs of health services [60], length of stay [48, 50, 60, 63], or total days spent in psychiatric inpatient treatment [65]. Boege et al. found significantly lower total healthcare costs of their “home treatment brings inpatient treatment outside” discharge intervention compared to usual care, factoring in length of stay, costs of hospitalization, therapy, and services [60]. Ougrin et al. used the CGAS and quality-adjusted life-years to evaluate cost effectiveness, calculating a probability of at least 50% that a supported discharge service is more cost effective than usual care, irrespective of willingness to pay, in addition to decreased inpatient days at six months following randomization [65]. Several other studies also measured length of stay as an outcome of their intervention [48, 50, 56, 63, 65]. Cameron et al. found that the Bridge Program resulted in reduced length of stay in the Young Adult Program (YAP) by almost 2 weeks (from 8.5 to 6.5 weeks) at the time of evaluation [50]. Doherty et al. reported that their bridging program was able to successfully deliver a treatment program restricting length of stay to 28 days, with a range of 1–71 days [63].

## Discussion

We identified 17 peer-reviewed articles and two grey literature documents for the review the components and settings of existing discharge interventions for inpatient children and adolescents in CAMHS, and their outcomes. All articles and documents discussed the promise and benefit of discharge interventions. Many interventions featured multiple components which assisted the discharge process for youth being discharged from inpatient CAMHS, including, a thorough assessment of the child’s needs [48, 49, 60, 62, 64], tailoring resources and services to needs and preferences [47, 48, 50, 58–60, 63–65], hospital discharge planning [55, 57, 58, 61, 63, 64], community linkage [47, 49–53, 58, 63], family education and support [47, 53, 54, 56, 58, 61, 63, 64], and a protocol in place for follow-up [49, 51, 52, 54, 59, 62, 64]. These discharge interventions demonstrated positive improvement in patient experiences or health outcomes [47–65]. Outcomes were identified across the dimensions of the Triple Aim, with the majority focused on population health outcomes.

This review highlights variability in the format, structure, and content of discharge interventions for inpatient youth attending CAMHS. The transitional discharge programs described by Furedy et al. [51] and Wasylenki et al. [49] were not specifically limited to pediatric populations and younger participants may have therefore been subjected to topics less applicable or relevant for their post-discharge experience. However, the majority of included studies described interventions catered towards a pediatric or youth population with a range of mental health comorbidities at intake. The length of interventions ranged from over the patient’s admission course, time of discharge, and several weeks-months of post-discharge support. The length of interventions varied depending on individual patient need. The diversity of the health professionals providing these interventions underscores the multidisciplinary nature of discharge planning—a process that involves a comprehensive understanding of patient and family needs. Existing literature on discharge interventions highlight the important role of discharge interventions in transitioning and preparing patients for life beyond the hospital [2, 24, 27, 66–69]. This review provides a better understanding of how discharge interventions improve pediatric outcomes. Specifically, results of the reviewed documents reported interventions to be beneficial in improving patient clinical functioning and self-sufficiency, caregiver skills, post-discharge service attendance, as well as minimizing societal costs [47–51, 53–56, 58, 60–65]. This is reflective of present literature, as highlighted in the article by Fontanella et al., who discussed the impact of discharge planning and timely aftercare on effectiveness of inpatient care and reducing readmissions [19]. The outcomes of this review also suggest discharge interventions were relevant and appreciated by the patient and their families, which may further support their reintegration to community post-discharge.

The interventions described in this scoping review were organized using existing frameworks that were created for different populations and settings. For instance, Hansen et al.’s [43] taxonomy of interventions using the three domains (i.e., pre-discharge, post-discharge, bridging interventions) was originally created to evaluating studies reducing rehospitalization within 30 days, and most of the studies included tested a single-component intervention. It proved challenging to adopt for the CAMHS discharge interventions included in this review, given the multicomponent nature of some discharge interventions. Bridging interventions often featured numerous different components that would have taken place before and/or after discharge (e.g., discharge planning, patient education). It should be noted that the taxonomy used was merely used to broadly categorize discharge interventions and that there exists a diverse range of interventions within each individual domain itself. The development of future standardized protocols or frameworks for discharge interventions from CAMHS may contribute towards a more refined analysis of intervention components.

The features and components identified in the discharge interventions reviewed share a number of similarities with the effective discharge planning framework components identified by Yam et al. [70]. Their delphi study identified the needs of a structured, systematic, coordinated hospital discharge system to ensure smooth transition from hospital to community [70]. Yet, our review also highlighted the lack of standardized discharge protocols and subsequent evaluation, and that research to implement such frameworks is still in its early stages [31, 65, 70]. A report by Health Quality Ontario promotes standardization for transitional care of complex patients [71]. The document “Adopting a Common Approach to Transitional Care Planning: Helping Health Links Improve Transitions and Coordination of Care” outlines discharge principles in three categories of transitional care: pre-transition practices, transition planning practices, and assessing post-transition risk and activating post-transition follow-up [71]. These principles align with our scoping review results—a priority on individualized, patient-centered care that also includes patient and caregiver involvement, coordination of continuing care to other resources or services, community relationships, and optimized timing and location of health care services.

The findings of this review provide the foundation for the engagement of stakeholders (i.e., youth, families, and clinicians) to determine clinically meaningful outcomes by which to evaluate transition interventions. What is considered “clinically meaningful” may be variable upon the perspective but generally takes into account the factors, condition, population, and benefits and risks of present interventions [72, 73]. Such outcomes for this pediatric population may include other patient-oriented factors impacting long-term functioning, relationships, or quality of life. The transition from inpatient to outpatient care in CAMHS has been highlighted as a complex process, with timely care related to multiple patient-, hospital-, and community-level characteristics [74]. As described by Fontanella et al., having previous experience of outpatient mental health care or connections with mental health providers is a strong predictor of linkage to timely outpatient care [74]. Discharge interventions reported in this review described a designated role or program that coordinated and advocated for such relationships with outpatient care and within their community [50, 58, 59].

This review may support the design and implementation of effective discharge interventions for children and youth and encourages a critical examination of present initiatives for possible innovation. It may also provide the necessary foundation and scope for future research involving discharge interventions for CAMHS inpatients.

### Future directions and implications

Limited guidelines and protocols in approaching discharge interventions may contribute to significant heterogeneity among the clinical sample, settings, intervention, and outcomes. A number of identified articles were descriptive in nature and lacked proper research design, which unfortunately limited the depth of analysis and conclusions drawn. Moreover, it precluded the authors’ abilities to conduct a deeper systematic review of the interventions identified. Differing definitions of a “discharge intervention” among studies and articles created further ambiguity during analysis. Future studies thus ought to be clearer as to what the discharge process entails and how decisions about discharge are made. Literature searches for discharge interventions may also be adapted to focus on criteria excluded in this review, such as emergency department settings, or for populations with developmental disabilities. These populations may have unique additional supports and resources and warrant a separate search and scoping review.

Researchers may also explore more rigorous methods (e.g., randomised controlled trials) to evaluate the impact of discharge interventions. Future research is needed to understand the elements of a good discharge intervention design, given that existing literature is limited in this area, particularly from the point of view of patients and/or caregivers. Integrating both discharge intervention elements and post-discharge timing elements may also effectively minimize adverse events once out of hospital and could be better examined under developing frameworks of discharge optimization [75].

Ultimately, our findings encourage a more rigorous evaluation of discharge interventions and the tools used to define and assess discharge interventions for children and youth. Researchers would benefit from increased collaboration with stakeholders, particularly patients and their caregivers, and including their input in both the design and evaluative stages of these projects [76].

### Limitations

This review has a number of limitations. First, the literature search was limited to the English language, which may have excluded articles that would have otherwise met eligibility criteria. Secondly, many interventions described a broader population, often describing a wide ranging population without providing further details to the sample’s age distribution or describing a “youth” or “school-aged population” without formally or explicitly stating the ages of the sample [49, 53, 54]. Consequently, the research team was required to make assumptions that these were pediatric populations. Thirdly, among the discharge interventions described in this review, the majority included were descriptive in nature. While there are some studies with promising findings, only a few of these have been evaluated, and even fewer in a controlled setting. Additionally, we faced difficulties in discerning which interventions described were truly “discharge” interventions, owing to the variability of intervention descriptions. Several authors provided limited detail on their discharge protocol or how discharge decisions were made, which created challenges for the team when trying to organize and group interventions. More dated studies of this review provided poorer descriptions of population, study measures, or incomplete reporting of outcomes. Poor study population descriptors and broad study settings without subgroup analysis for different age groups or settings may mean that the stated outcomes may be less applicable to children and youth in the psychiatric setting.

## Conclusion

This scoping review presented evidence on the components and outcomes of discharge interventions from inpatient CAMHS settings. Common elements among interventions included risk assessment, individualized care, discharge preparation, community linkage, psychoeducation, and follow-up support. Promising outcomes included positive patient and caregiver satisfaction, improved patient health outcomes, and increased cost effectiveness. Despite the diversity among populations, goals, and outcomes of interventions, these components facilitate successful discharge processes and reduce likelihood of readmissions among children and adolescents with psychiatric illnesses. Present findings are limited by across-study heterogeneity, inadequate reporting, and lack of controlled study design. Findings may be used to promote the need for a deeper systematic analysis of the outcomes of discharge interventions and a further evaluation of the elements supporting successful CAMHS discharges.
